# Supplementary material for: Haplotype editing with CRISPR/Cas9 as a therapeutic approach for dominant-negative missense mutations in NEFL
Source: bioRxiv. 2024 Dec 22:2024.12.20.629813. Preprint. [Version 1] doi: 10.1101/2024.12.20.629813 (PMC11702708; doi:10.1101/2024.12.20.629813)
Supplement: Supplement 1 [file media-1.pdf]

Table 1: Genomic off-target analysis

N98S gRNA

#	chr	start (hg38)	End (hg38)	Strand	CFD score	Description	Results: NF-P3 FS	Results: NF-P3 HDR
1	1	148316436	148316458	+	0.7143	intergenic: RP11-495P10.8/RP11-495P10.5/RP11-495P10.7-RP11-495P10.3	Sequencing Failed	Sequencing Failed
2	12	5752303	5752303	-	0.4412	intron: ANO2	PCR failed	PCR failed
3	10	75661072	75661094	-	0.3956	intergenic: RP11-310J24.3-RP11-367B6.2	No mutations	No mutations
4	20	48137627	48137649	+	0.3652	intergenic: AL139351.1-LINC00494	No mutations	No mutations
5	18	39020019	39020041	-	0.3649	intergenic: RN7SKP182-RNU6-706P	No mutations	No mutations
6	4	3605528	3605550	-	0.3563	intergenic: LINC00955-RP3-368B9.2	No mutations	No mutations
7	9	65426450	65426472	+	0.3563	intergenic: FOXD4L5-Y_RNA	No mutations	No mutations
8	8	1925519	1925541	-	0.3545	intron: ARHGEF10	No mutations	No mutations
9	13	111494882	111494904	+	0.3254	intergenic: TEX29-RP11-65D24.2	No mutations	No mutations
10	8	120892179	120892201	-	0.3196	intergenic: RP11-713M15.2/SNTB1-RP11-369K17.1	No mutations	No mutations
11	9	14223924	14223946	+	0.2790	intron: NFIB	No mutations	No mutations

L1.1G and R1.1G

#	gRNA	chr	start (hg38)	End (hg38)	Strand	CFD score	Description	Results: NF-P3 ex	Results: NF-P3 inv
1	R1.1G	10	78632335	78632357	-	0.49777778	exon:KCNMA1	No mutations	No mutations
2	R1.1G	3	40733428	40733450	+	0.3799606	intergenic:RP11-528N21.1-HMGN2P24	No mutations	No mutations
3	R1.1G	5	8208980	8209002	+	0.34821429	intergenic:RP11-215I16.1-RP11-480D4.1	No mutations	No mutations
4	R1.1G	6	18441294	18441316	+	0.31533434	intron:RNF144B	No mutations	No mutations
5	R1.1G	8	48092200	48092222	+	0.26848498	intergenic:NDUFA5P12-RP11-1134I14.4	No mutations	No mutations
6	R1.1G	11	79198180	79198202	-	0.38787879	intergenic:TENM4-RP11-258O13.1	No mutations	No mutations
7	R1.1G	12	34271299	34271321	+	0.38956522	intergenic:RP11-847H18.3-RP11-313F23.3	Cell line has a 5 bp variant. No other mutations detected.	Cell line has a 5 bp variant. No other mutations detected.
8	R1.1G	4	72155681	72155703	+	0.38181818	intergenic:SLC4A4-RP11-1J11.1	No mutations	No mutations
9	R1.1G	5	133553321	133553343	+	0.36363636	intergenic:CTD-2410N18.4/CDKL3/PPP2CA-PPP2CA	No mutations	No mutations
10	R1.1G	6	68788813	68788835	-	0.32223776	intergenic:RP11-542F9.1-RP11-406O16.1	No mutations	No mutations
11	R1.1G	4	189766672	189766694	-	0.30198176	intergenic:RP11-756P10.2-RP11-818C3.1	No mutations	No mutations

L1.1T and R1.1C

#	gRNA	chr	start (hg38)	End (hg38)	Strand	CFD score	Description	Results: NF-P8 ex	Results: NF-P8 inv
1	L1.1T	5	2501128	2501150	+	0.4982699	intergenic:Y_RNA-	No mutations	No mutations
2	L1.1T	11	43929675	43929697	-	0.29658922	intergenic:ALKBH3-ALKBH3-AS1	No mutations	No mutations
3	L1.1T	13	102135513	102135535	+	0.27731092	intron:ITGBL1	No mutations	No mutations
4	R1.1C	12	57160521	57160543	+	0.46928467	intron:HSD17B6	No mutations	No mutations
5	R1.1C	10	92818471	92818493	-	0.45389474	intergenic:DDX18P6-LINC00502	No mutations	No mutations
6	R1.1C	3	156692992	156693014	-	0.35	intergenic:LEKR1-KLF3P2	No mutations	No mutations
7	R1.1C	3	80744740	80744762	-	0.27575758	intergenic:RP11-481N16.1-RP11-47P18.1	PCR failed	PCR failed
8	R1.1C	3	4047631	4047653	+	0.44426162	intergenic: PNPT1P1-SUMF1	No mutations	No mutations
9	R1.1C	2	57033442	57033464	+	0.56384439	intergenic:AC008173.1-snoU13	Homozygous alternate SNP. No other mutations detected.	Homozygous alternate SNP. No other mutations detected.

Table 2: gRNA sequences

sgRNA Name	Target	Sequence	Allele Specific (AS) or Biallelic (BA)
N98S	N98S	agctggcgaagcggtcactg	AS
E396K	E396K	caggaaacUcUUggaaggcA	AS
L1.1G	rs2979688	GATCACGGCACGCCGGCCAG	AS
L1.2G	rs2979688	AGCGCGCTGCCCCCACTGGC	AS
L2T	rs2979687	TCACGGGGTCTGGGCAATGC	AS
L3C	rs2976437	TCTACATATGGGTAATTGGG	AS
L4A	rs2976436	ACCCATATGTAGATGAAGCA	AS
R1.1G	rs2979701	TCTGTGATAGGTTAGTGTAG	AS
R1.2G	rs2979701	CTGTGATAGGTTAGTGTAGA	AS
R2.1T	rs2979699	TACCAGGGTGACTGGAGTGC	AS
R2.2T	rs2979699	TTCCAGCACTCCAGTCACCC	AS
R3G	rs2976441	GCTTAAATGTCATTCTCTAA	AS
L1.1T	rs2979688	GATCACGGCACGCCGTCCAG	AS
L1.2T	rs2979688	AGCGCGCTGCCCCCACTGGA	AS
R1.1C	rs2979701	TCTGTGATAGGTTAGTCTAG	AS
R1.2C	rs2979701	CTGTGATAGGTTAGTCTAGA	AS
BA1	Intron 1	gcagctttaatgcggaacgc	BA
BA2	Intron 1	CCTTTATTTAGTAGGTAGAC	BA

Table 3: ssODN sequences for excisions

**L1.1G**	**BA**	CTTGGCTGCAGCAGCGCGCTGCCCCCACTGTTCCGCATTAAAGCTGCCCAGCCCTTGTTG
**L1.2G**	**HW15**	CCTTGGCTGCAGCAGCGCGCTGCCCCCACTTTCCGCATTAAAGCTGCCCAGCCCTTGTTG
**L1.2G**	**R1.1G**	CCTTGGCTGCAGCAGCGCGCTGCCCCCACTCACTAACCTATCACAGAGTTATAGTGAGAA
**L1.1G**	**R1.1G**	CCTTGGCTGCAGCAGCGCGCTGCCCCCACTCACTAACCTATCACAGAGTTATAGTGAGAA
**BA**	**R1.1G**	GGGAGTGTGCTCCGTGCTGCTGCACCGGCGCACTAACCTATCACAGAGTTATAGTGAGAA
**L1.1T**	**BA**	CTTGGCTGCAGCAGCGCGCTGCCCCCACTGTTCCGCATTAAAGCTGCCCAGCCCTTGTTG
**L1.2T**	**BA**	CCTTGGCTGCAGCAGCGCGCTGCCCCCACTTTCCGCATTAAAGCTGCCCAGCCCTTGTTG
**L1.2T**	**R1.1C**	CCTTGGCTGCAGCAGCGCGCTGCCCCCACTGACTAACCTATCACAGAGTTATAGTGAGAA
**L1.1T**	**R1.1C**	CCTTGGCTGCAGCAGCGCGCTGCCCCCACTGACTAACCTATCACAGAGTTATAGTGAGAA
**BA**	**R1.1C**	GGGAGTGTGCTCCGTGCTGCTGCACCGGCGGACTAACCTATCACAGAGTTATAGTGAGAA

Table 4: ddPCR assays for excision and inversion frequency

exc / inv	R1.1	R1.2	R2.1	R2.2	R3
**L4**	2 / B	2 / B	3 / C	3 / C	4 / D
**L3**	2 / B	2 / B	3 / C	3 / C	4 / D
**L2**	6 / F	14 / N	12 / L	12 / L	10 / J
**L1.2**	7 / G	13 / M	11 / K	11 / K	9 / I
**L1.1**	8 / G	13 / M	11 / K	11 / K	9 / I

Excision Assays

Assay Number	F primer	R primer	Probe
2	HW_ddXR_79_F	HW_ddXR_63_R	HW79
3	HW_ddXR_79_F	HW_ddXR_55_R	HW79
4	HW_ddXR_79_F	HW_ddXR_53_R	HW79
6	HW_ddXR_74_F	HW_ddXR_63_R	HW63
7	HW_ddXR_69_F	HW_ddXR_63_R	HW63
8	HW_ddXR_72_F	HW_ddXR_63_R	HW63
9	CM_ddXR_4790_F	CM_ddXR_11390_R	HW69
10	CM_ddXR_4590_F	CM_ddXR_11390_R	HW74
11	CM_ddXR_4790_F	CM_ddXR_11270_R	HW69
12	CM_ddXR_4590_F	CM_ddXR_11270_R	HW74
13	CM_ddXR_4890_F	CM_ddXR_10970_R	HW63
14	CM_ddXR_4650_F	CM_ddXR_10970_R	HW63

Inversion Assays

Assay Letter	F primer	R primer	Probe
B	HW_ddXR_79_F	CM_iddXR_63_R	HW79
C	HW_ddXR_79_F	CM_iddXR_55_R	HW79
D	HW_ddXR_79_F	CM_iddXR_53_R	HW79
F	CM_iddXR_74_F	HW_ddXR_63_R	HW63
G	CM_NEFL_4996_R	HW_ddXR_63_R	HW63
I	CM_iddXR_11250_F	CM_iddXR_11430_R	HW13
J	CM_ddXR_4590_F	CM_iddXR_4840_R	HW74
K	CM_iddXR_11110_F	CM_iddXR_11310_R	HW13
L	CM_ddXR_4590_F	CM_iddXR_4800_R	HW74
M	CM_iddXR_10800_F	CM_iddXR_10950_R	HW63
N	CM_iddXR_10810_F	CM_iddXR_10950_R	HW63

Table 5: Primer and Probe Sequneces for ddPCR assays for excision and inversion frequency

Primer Sequences

Name	Sequence (5' → 3')
CM_ddXR_4790_F	ggcatgggatctcagagaaa
CM_ddXR_11390_R	caaagaatttgacccactagaagag
CM_ddXR_4590_F	gaggttcctggtgggaaa
CM_ddXR_11270_R	aaagatgagtgctccagaaa
CM_ddXR_4890_F	cgcagaatcctcgcctt
CM_ddXR_10970_R	ccctgggagaagggttaga
CM_ddXR_4650_F	gaggtgacgggatacagaaa
HW_ddXR_79_F	tatgcagactcacacacactg
HW_ddXR_72_F	GCAGAATCCTCGCCTTGG
HW_ddXR_74_F	GGGCAACTTAAGGATCCAAGT
HW_ddXR_63_R	gtggtggcagtataaattgaaaga
HW_ddXR_55_R	ATCCTGTGACAGATGGGAGAA
HW_ddXR_53_R	TTCAAAGAATTTGACCCACTAGAAG
CM_iddXR_74_F	GCTCAGAGGGCCCTGATTTT
CM_iddXR_63_R	CTGTGGTCAGTGCCCCTTTT
CM_iddXR_55_R	CTGCCTAGTGCTGACTCCTG
CM_iddXR_53_R	CTCCACTTCCAGCACTCCAG
CM_NEFL_4996_R	ccgttctgccacccctattt
CM_iddXR_11250_F	gaggatggatggctgtgtg
CM_iddXR_11110_F	gaggatggatggctgtgtg
CM_iddXR_10800_F	tatttatacgccgggaggct
CM_iddXR_10810_F	ccctcactcatttcccttctg
CM_iddXR_11430_R	agatgctaatggcaagaatcaa
CM_iddXR_4840_R	ctcccatctgtcacaggattt
CM_iddXR_11310_R	aatctgaagggtcagtaggaac
CM_iddXR_4800_R	ctgactcctgcctagttctcta
CM_iddXR_10950_R	gtggtggcagtataaattgaaagat

Probe Sequences

Name	Sequence (5' → 3')
HW79	TGAGGTTTGCAGGGAGCAGGTTAA
HW63	ACACCTCCATGTCTTAGATCCTTCCACA
HW13	caggctgcgtcagg
HW69	tctgagcaaagtggaaaggacgacc
HW74	ctgcgaggtgacgggatacagaaa

Table 6: Antibodies

Name/Antigen	Host	Vendor/Catalog #	IF Concentration
NF-L	Rabbit	millipore<br>AB9568	1:1000 for CX7,<br>1:500 for Keyence
β-Tubulin III	Mouse	ThermoFisher<br>480011	1:250
HB9	Mouse	DSHB 81.5c10	1:200
Anti-rabbit<br>Alexa Fluor 488	Goat	Invitrogen A11034	1:500
Anti-mouse<br>Alexa Fluor 594	Goat	Invitrogen A11032	1:500

Table 7 : Assays for inversions and excisions

Excision Frequency			
**Guide Pair**	**F Primer**	**R Primer**	**Probe**
L1.1 - BA	PD_ddXR_72/73F_anchored	PD_ddXR_HW15_R	HW63
L1.1 - R1.1	HW_ddXR_72_F	HW_ddXR_63_R	HW63
BA - R1.1	HW_ddXR_15/63F	HW_ddXR_63_R	HW63

Inversion Frequency			
**Guide Pair**	**F Primer**	**R Primer**	**Probe**
L1.1 - BA	PD_ddXR_72/73F_anchored	PD_iddXR_HW15_R	HW63
L1.1 - R1.1	CM_NEFL_4996_R	HW_ddXR_63_R	HW63
BA - R1.1	HW_iddXR_15/63F	HW_ddXR_63_R	HW63

Excision Specificity via ddPCR			
**Guide Pair**	**F Primer**	**R Primer**	**Allele Discrimination Assay**
L1.1 - BA	NEFL_forASaroundrs2979685	PD_7984_R	rs2979685
L1.1 - R1.1	NEFL_forASaroundrs2979685	HW_ddXR_63_R	rs2979685
BA - R1.1	NEFL_forASaroundrs2979685	HW_ddXR_63_R	rs2979685

Inversion Specificity via ddPCR			
**Guide Pair**	**F Primer**	**R Primer**	**Allele Discrimination Assay**
L1.1 - BA	NEFL_forASaroundrs2979685	HW_NEFL_5740_F	rs2979685
L1.1 - R1.1	NEFL_forASaroundrs2979685	BMJS_NEFL_7758F	rs2979685
BA - R1.1	NEFL_forASaroundrs2979685	HW_ddXR_63_R	rs2979685

Table 8: Primer sequences for excision and inversion assays

Name	Sequence 5' → 3'
PD_ddXR_72/73F_anchored	CCCTCTGAGCAAAGTGGAAA
HW_ddXR_15/63F	TGTAGTCTGGGAGTGTGCT
HW_iddXR_15/63F	CCCAATTCCCACGTCTTCC
NEFL_forASaroundrs2979685	GGCTGTCGTGGAGTATGAGG
PD_ddXR_HW15_R	CAGGTCAGTAGAGAGCTGAT
HW_ddXR_63_R	GTGGTGGCAGTATAAATTGAAAGA
PD_iddXR_HW15_R	TGTAGTCTGGGAGTGTGCT
PD_7984_R	CTCCTCTTGGACATGGCTGG
HW_ddXR_72_F	GCAGAATCCTCGCCTTGG
CM_NEFL_4996_R	CCGTTCTGCCACCCCTATTT
HW_NEFL_5740F	TCGACAGCTTGATGGACGAA
BMJS_NEFL_7758F	TCCTGCTTGCCTTTGTGTTTAG

Table 9 : hNIL copy number 20x primer probe mixes

hNIL copy number ddPCR custom mixes			
	F Primer	R Primer	Probe
Neomycin Assay	catggctgatgcaatgcg	tcgcttggtggtcgaatg	cgcttgatccggctacctgcc
TRE3G Assay	tacggtgggcgcctataa	agtggtacggaaagttggtataag	agatcgcctggagcaattccacaa

Table 10: hNIL genotyping junction PCR primer sequences

Junction PCR Primers for hNIL CLYBL integration	
hNIL CLYBL 5' Junction F	CAGACAAGTCAGTAGGGCCA
hNIL CLYBL 5' Junction R	AGAAGACTTCCTCTGCCCTC
hNIL CLYBL 3' Junction F	CACCAGCAACCTGACGTTTT
hNIL CLYBL 3' Junction R	TTTTATAGGCGCCCACCGTA
CLYBL WT F	TGACTAAACACTGTGCCCCA
CLYBL WT R	AGGCAGGATGAATTGGTGGA

Post-Cre Junction PCR Primers	
CF28	CCTCAGCCCAGTTTCCACTTG
CF29	GGCTATGAACTAATGACCCCGT

Table 11 : Primer sequences for next generation sequencing

Name	Sequence 5' → 3' with illumina adapter sequence
BMJS_NGS_10733F	TCCCTACACGACGCTCTTCCGATCTcagctAgttctctttcagtattccctctcc
BMJS_NGS_10965R	GTTCAGACGTGTGCTCTTCCGATCTcagctAggttagagtggtggcagtataaa
BMJS_NGS_4810F	TCCCTACACGACGCTCTTCCGATCTcagctAccctctgagcaaagtggaaa
BMJS_NGS_5025R	GTTCAGACGTGTGCTCTTCCGATCTcagctAgaggatggatggctgtgtg
