# Supplementary material for: Haplotype editing with CRISPR/Cas9 as a therapeutic approach for dominant-negative missense mutations in NEFL
Source: bioRxiv. 2024 Dec 22:2024.12.20.629813. Preprint. [Version 1] doi: 10.1101/2024.12.20.629813 (PMC11702708; doi:10.1101/2024.12.20.629813)
Supplement: Supplement 4 [file NIHPP2024.12.20.629813v1-supplement-4.pdf]

bioRxiv preprint doi: https://doi.org/10.1101/2024.12.20.629813; this version posted December 22, 2024. The copyright holder for this preprint (which was not certified by peer review) is the author/funder, who has granted bioRxiv a license to display the preprint in perpetuity. It is made available under aCC-BY 4.0 International license.

SUPPLEMENTARY DATA

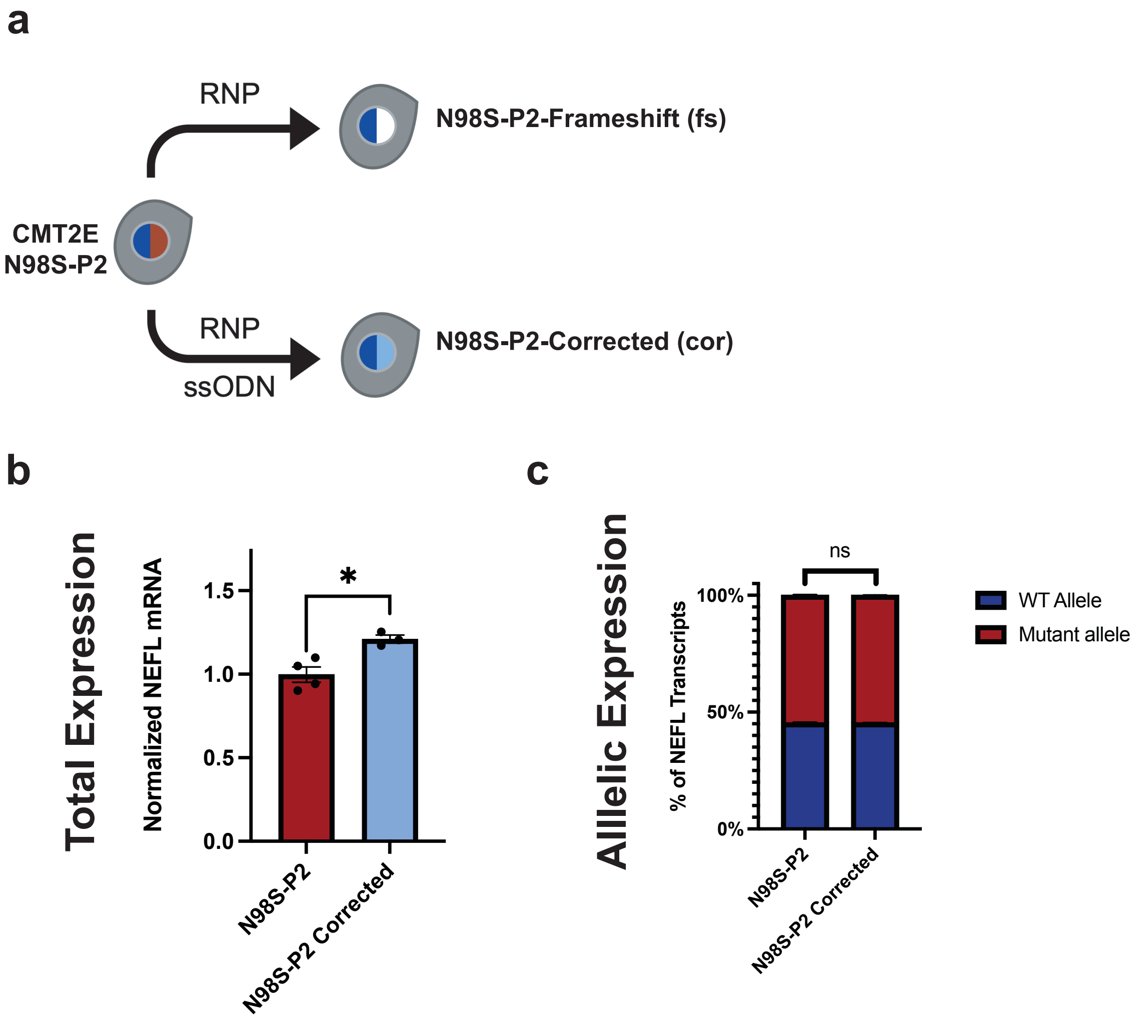

Figure S1: Derivation of clonal edited iPSC lines from the CMT2E N98S-P2 patient line **(a)** Transfection of N98S-P2 iPSC with N98S-specific HiFiCas9 RNP produced N98S-P2-fs (contains +1 indel at the mutation locus). Transfection of N98S-P2 iPSC with N98S-specific HiFiCas9 RNP plus single-strand oligonucleotide donor produced N98S-P2-cor (precise correction of N98S mutation with linked silent mutation to facilitate genotyping). Colored nuclei indicate *NEFL* genotype: dark blue = wild type, red = N98S mutant, white = frameshift (knockout), light blue = N98S-corrected (+ silent mutation). N98S-P2 and N98S-P2-cor were differentiated into i^{3}LMNs and RNA was extracted on day 7. **(b)** Total *NEFL* expression by quantitative RT-ddPCR, normalized to GAPDH. **(c)** Relative allelic expression by allele discrimination RT-ddPCR. Bar graphs represent mean +/- S.E.M. of biological replicates. * $p < 0.05$, ns $p > 0.05$.

bioRxiv preprint doi: https://doi.org/10.1101/2024.12.20.629813; this version posted December 22, 2024. The copyright holder for this preprint (which was not certified by peer review) is the author/funder, who has granted bioRxiv a license to display the preprint in perpetuity. It is made available under aCC-BY 4.0 International license.

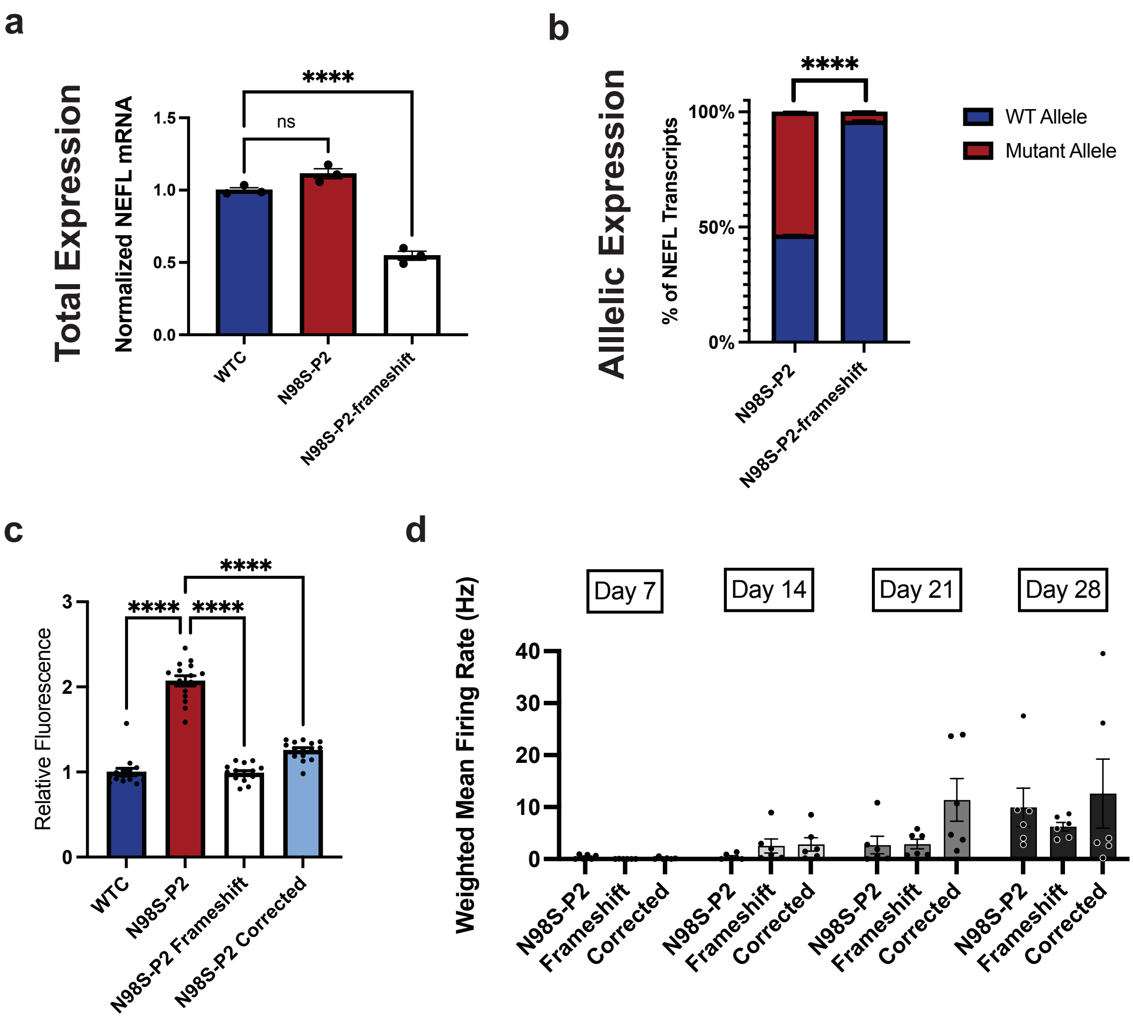

Figure S2: Frameshift editing in N98S-P2 i^{3}LMNs inactivates the mutant allele and rescues disease phenotype. N98S-P2 and N98S-P2-fs were differentiated into i^{3}LMNs and subsequently used for RNA extraction or immunofluorescent staining on day 7. **(a)** Total *NEFL* expression by quantitative RT-ddPCR, normalized to *GAPDH*. **(b)** Relative allelic expression by allele discrimination RT-ddPCR. **(c)** Mean NF-L fluorescence intensity in the cell bodies of iPSC-derived motor neurons at day 7. **(d)** Multi-electrode array was conducted to measure spontaneous action potentials in N98S-P2, and in corrected and frameshift lines. Spontaneous electrical activity was measured at days 7, 14, 21, and 28. Each data point represents the mean weighted firing rate from one biological replicate. Two-way ANOVA demonstrated a statistically significant effect of the differentiation day ($p < 0.001$) but no significant difference between cell lines ($p > 0.05$). Bar graphs represent mean +/- S.E.M. of biological replicates. **** = $p < 0.0001$, ns $p > 0.05$.

bioRxiv preprint doi: https://doi.org/10.1101/2024.12.20.629813; this version posted December 22, 2024. The copyright holder for this preprint (which was not certified by peer review) is the author/funder, who has granted bioRxiv a license to display the preprint in perpetuity. It is made available under aCC-BY 4.0 International license.

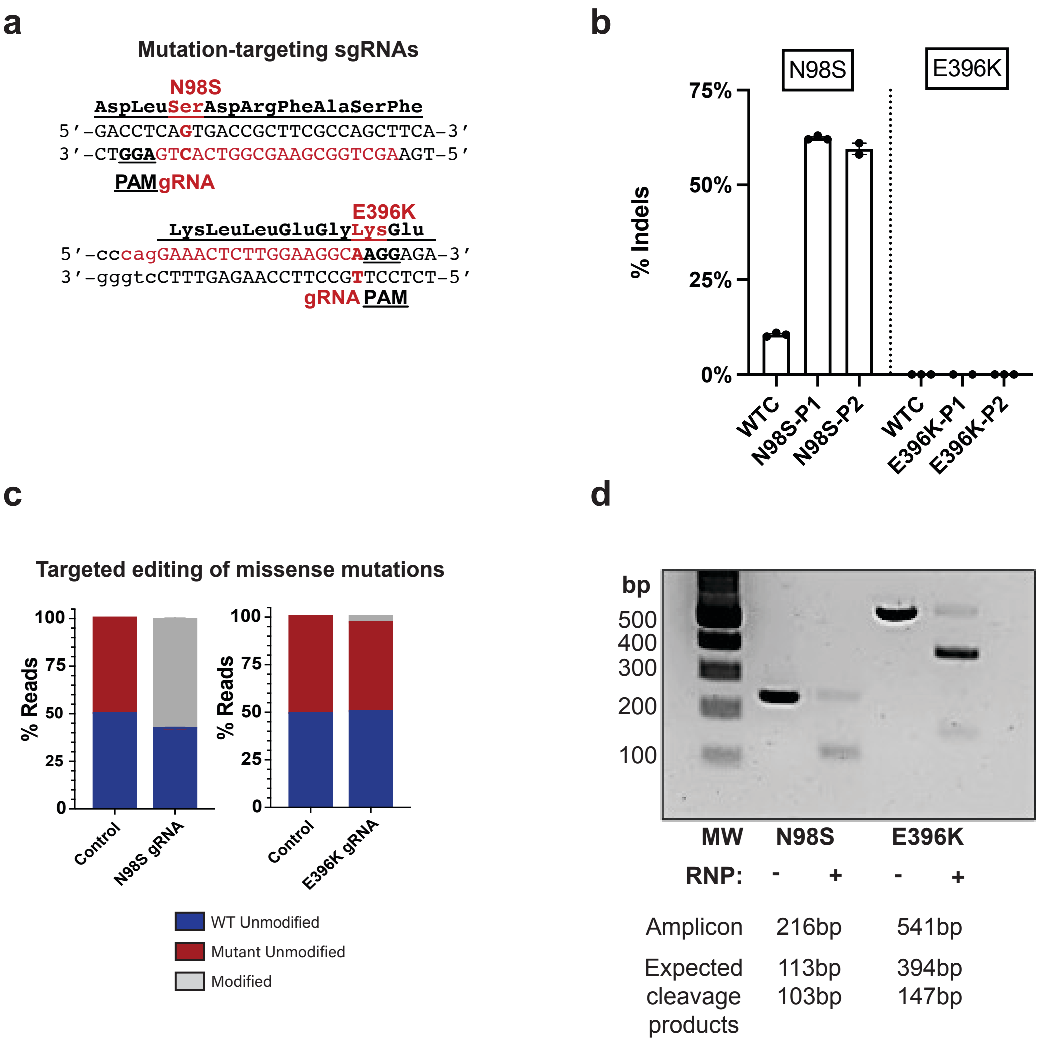

Figure S3: Comparison of the editing and nuclease activity of gRNAs targeting the N98S and E396K mutations. **(a)** Sequences of N98S and E396K mutation-targeting gRNAs (red) with associated PAM sequences (underlined). **(b)** Indel editing efficiency of N98S and E396K mutation-specific gRNAs in multiple patient backgrounds by Sanger sequencing and ICE analysis. Bar graphs represent mean +/- S.E.M of replicate transfections. **(c)** NGS amplicon sequencing of N98S and E396K targeted editing in N98S-P2 and E396K-P2 iPSCs. Modified reads contain indels at the target site, WT and mutant unmodified indicate reads without indels on the WT and mutant alleles, respectively. **(d)** PCR amplicons spanning the N98S or E396K mutations were generated from N98S-P2 and E396K-P2 gDNA, respectively, followed by incubation with corresponding N98S or E396K mutation-specific gRNA and HiFiCas9 RNP. Gel electrophoresis identifies the expected cleavage products. MW = molecular weight DNA ladder.

bioRxiv preprint doi: https://doi.org/10.1101/2024.12.20.629813; this version posted December 22, 2024. The copyright holder for this preprint (which was not certified by peer review) is the author/funder, who has granted bioRxiv a license to display the preprint in perpetuity. It is made available under aCC-BY 4.0 International license.

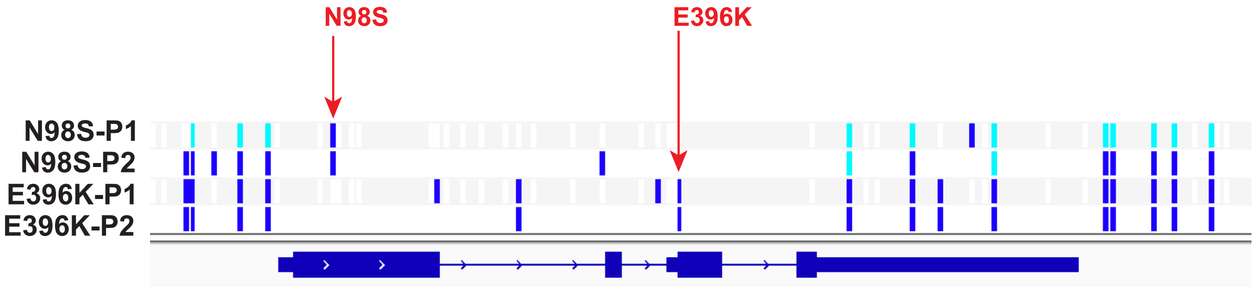

Figure S4: Panel of patient cell lines with variants flanking the *NEFL* coding region. Whole genome sequencing was performed from patient-derived iPSC. Dark blue lines indicate heterozygous variants, light blue lines indicate homozygous variants. Causative missense mutations are annotated with red arrows. Images generated from vcf files using Integrative Genomics Viewer[47].

bioRxiv preprint doi: https://doi.org/10.1101/2024.12.20.629813; this version posted December 22, 2024. The copyright holder for this preprint (which was not certified by peer review) is the author/funder, who has granted bioRxiv a license to display the preprint in perpetuity. It is made available under aCC-BY 4.0 International license.

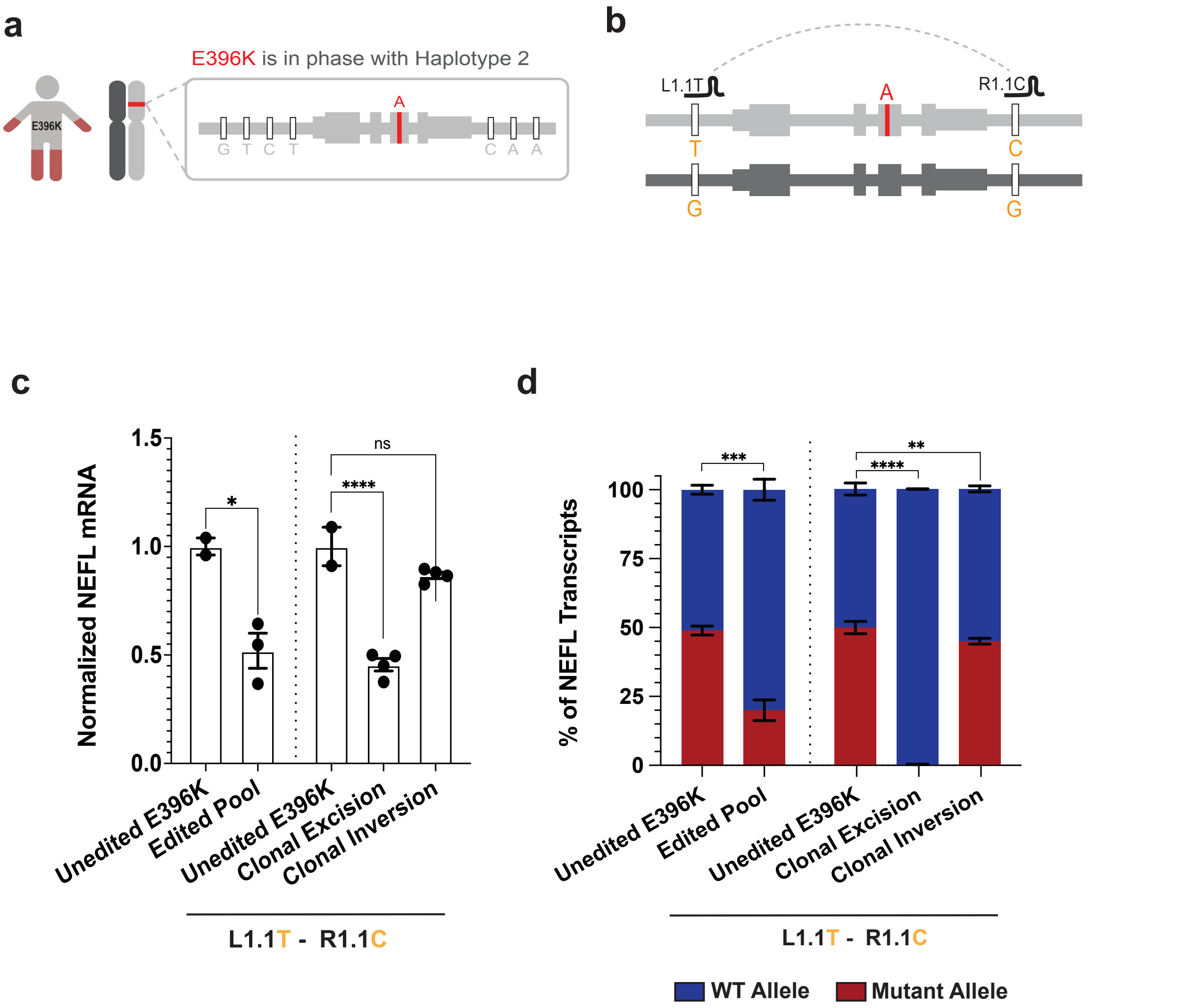

Figure S5: Analysis of *NEFL* gene expression after haplotype editing in E396K-P2 i³LMNs. **(a)** Schematic of phasing of E39K mutation with Haplotype 2 **(b)** Schematic of SNP-targeting guide pair for E396K haplotype editing. Edited and unedited iPSCs from E396K-P2 were differentiated into i³LMNs, and RNA was extracted on Day 7. **(c)** Total *NEFL* expression was measured by quantitative RT-ddPCR relative to *GAPDH* and normalized to the unedited control in. **(d)** Relative expression of wildtype and mutant alleles was measured by allele discrimination ddPCR. Bar graphs represent mean +/- S.E.M of biological replicates. * $p < 0.05$, ** $p < 0.01$, *** $p < 0.001$, **** $p < 0.0001$, ns $p > 0.05$.

bioRxiv preprint doi: https://doi.org/10.1101/2024.12.20.629813; this version posted December 22, 2024. The copyright holder for this preprint (which was not certified by peer review) is the author/funder, who has granted bioRxiv a license to display the preprint in perpetuity. It is made available under aCC-BY 4.0 International license.

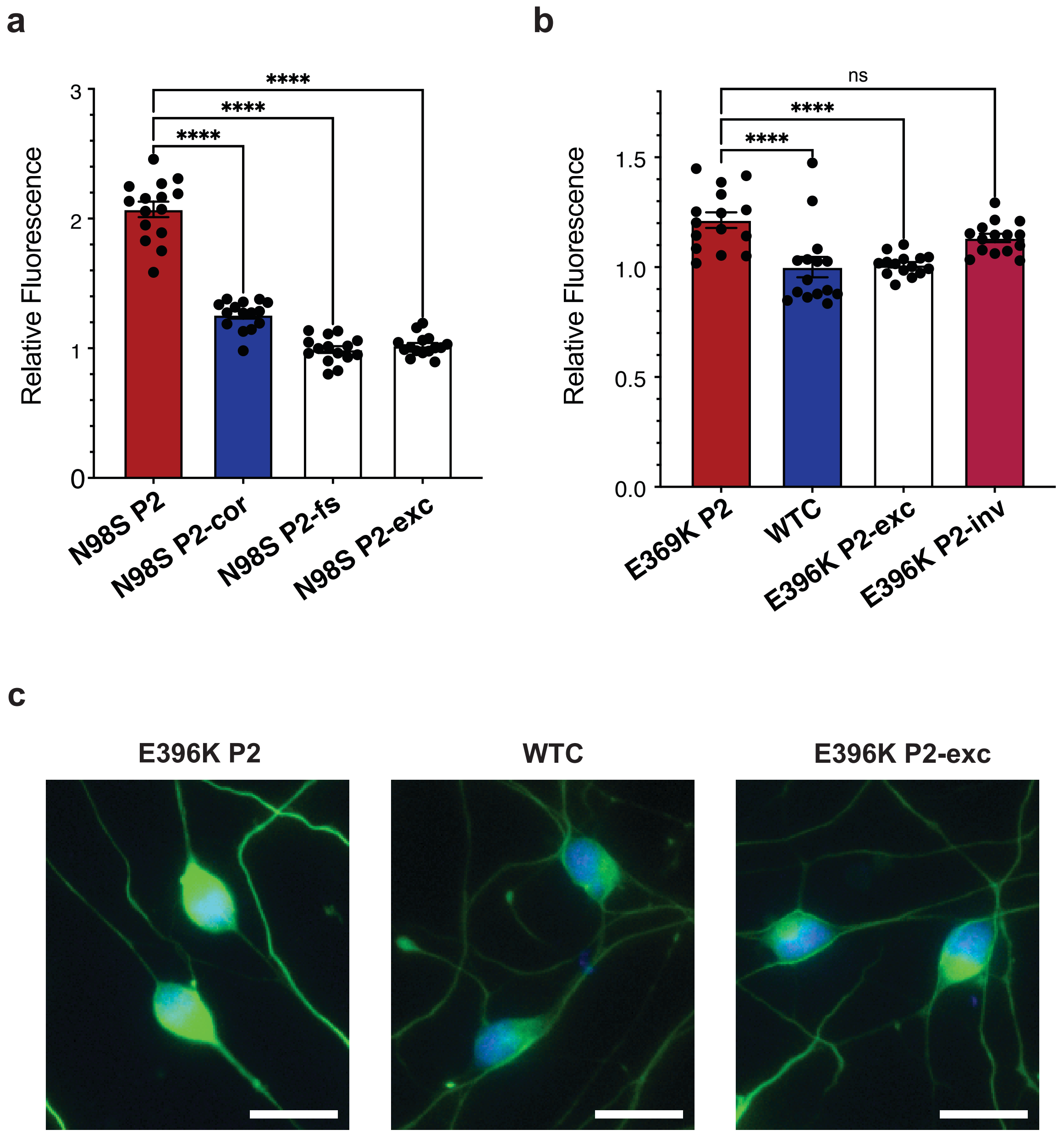

Figure S6: NF-L accumulation in cell bodies of control and edited i^{3}LMNs. Quantification of mean NF-L fluorescence intensity in inferred cell bodies in **(a)** N98S-P2 and **(b)** E396K-P2 i^{3}LMNs at day 7. Each data point is the mean NF-L intensity calculated across five images per replicate. Bar graphs represent mean +/- S.E.M of biological replicates. **** $p < 0.0001$, ns $p > 0.05$. **(c)** Representative images of E396K-P2 i^{3}LMNs stained with anti-NF-L (green) and anti-HB9 (blue). Scale bars = 20 µM.

bioRxiv preprint doi: https://doi.org/10.1101/2024.12.20.629813; this version posted December 22, 2024. The copyright holder for this preprint (which was not certified by peer review) is the author/funder, who has granted bioRxiv a license to display the preprint in perpetuity. It is made available under aCC-BY 4.0 International license.

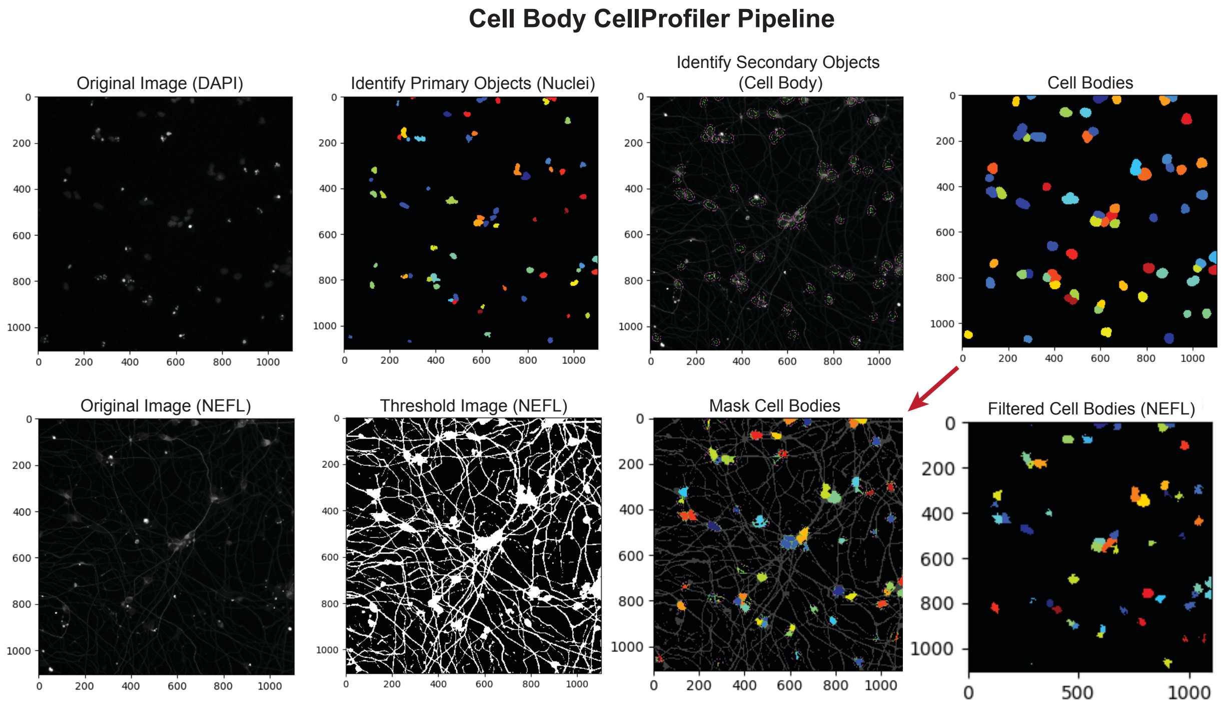

Figure S7: CellProfiler pipeline for measuring NF-L intensity in i^{3}LMN cell bodies. Nuclei (primary objects) were identified from the DAPI-stained image channel. Cell bodies (secondary objects) were inferred by extending the area of DAPI+ signal by 10 pixels. The NF-L-stained image channel was thresholded and masked with these inferred cell bodies (red arrow). The cell bodies were then filtered by shape and compactness to remove any incorrectly identified objects. The mean intensity in the cell body-masked NF-L-stained image channel was measured to quantify NF-L within cell bodies.

bioRxiv preprint doi: https://doi.org/10.1101/2024.12.20.629813; this version posted December 22, 2024. The copyright holder for this preprint (which was not certified by peer review) is the author/funder, who has granted bioRxiv a license to display the preprint in perpetuity. It is made available under aCC-BY 4.0 International license.

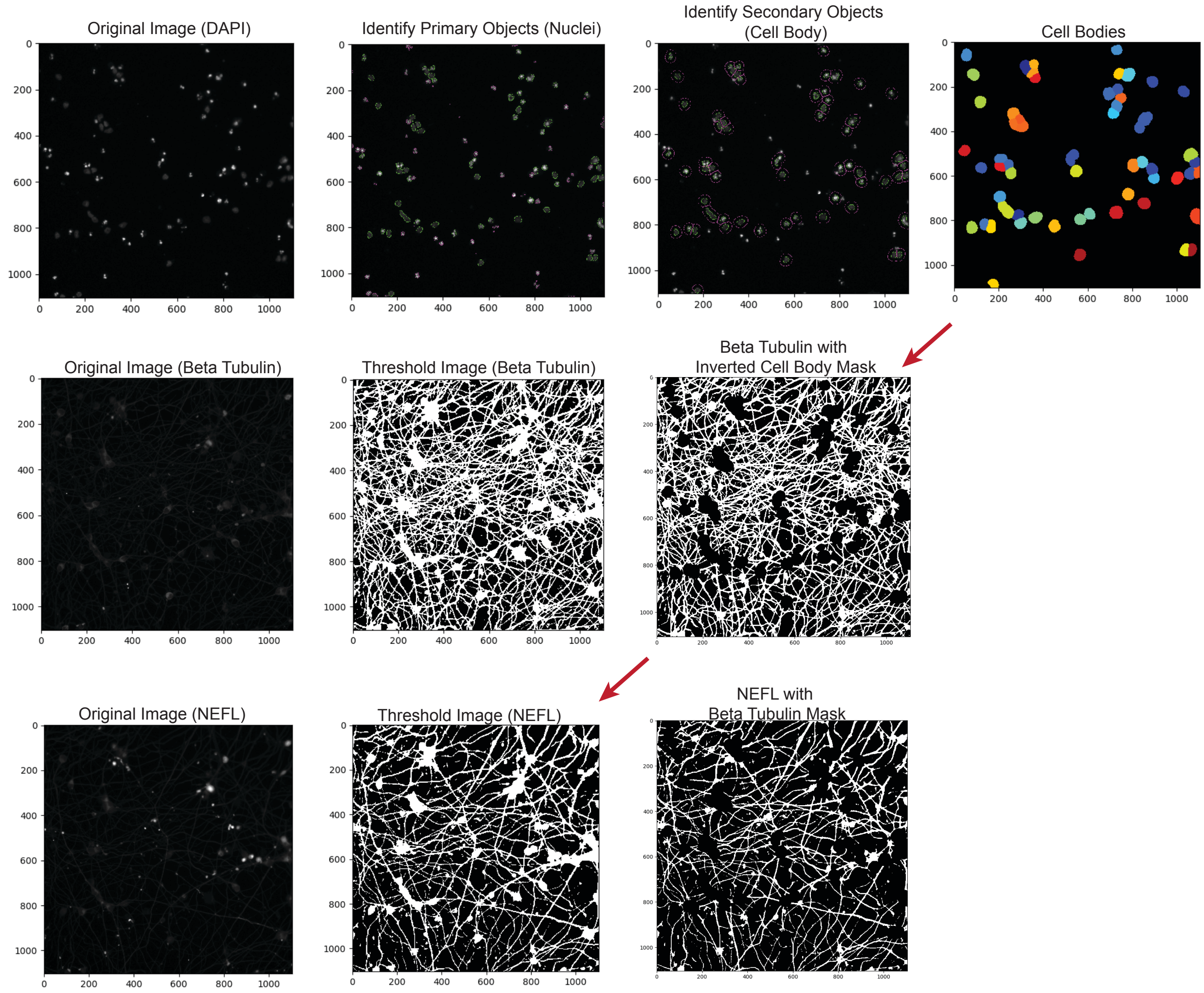

Figure S8: CellProfiler pipeline for measuring NF-L intensity in i^{3}LMN neurites. Nuclei (primary objects) were identified from the DAPI-stained image channel. Cell bodies (secondary objects) were inferred by extending the area of the DAPI+ signal by 15 pixels. The beta3-tubulin-stained image channel was thresholded, and an inverted cell body mask was applied to exclude signal within the cell bodies and define the region encompassed by neurites. The NF-L-stained image channel was then thresholded and masked with the beta3-tubulin neurite image. Red arrows indicate the application of masks to images in other channels. Total intensity in the neurite-masked NF-L-stained image channel was calculated and divided by the beta3-tubulin-positive neurite area to normalize for variation in neurite density.

bioRxiv preprint doi: https://doi.org/10.1101/2024.12.20.629813; this version posted December 22, 2024. The copyright holder for this preprint (which was not certified by peer review) is the author/funder, who has granted bioRxiv a license to display the preprint in perpetuity. It is made available under aCC-BY 4.0 International license.

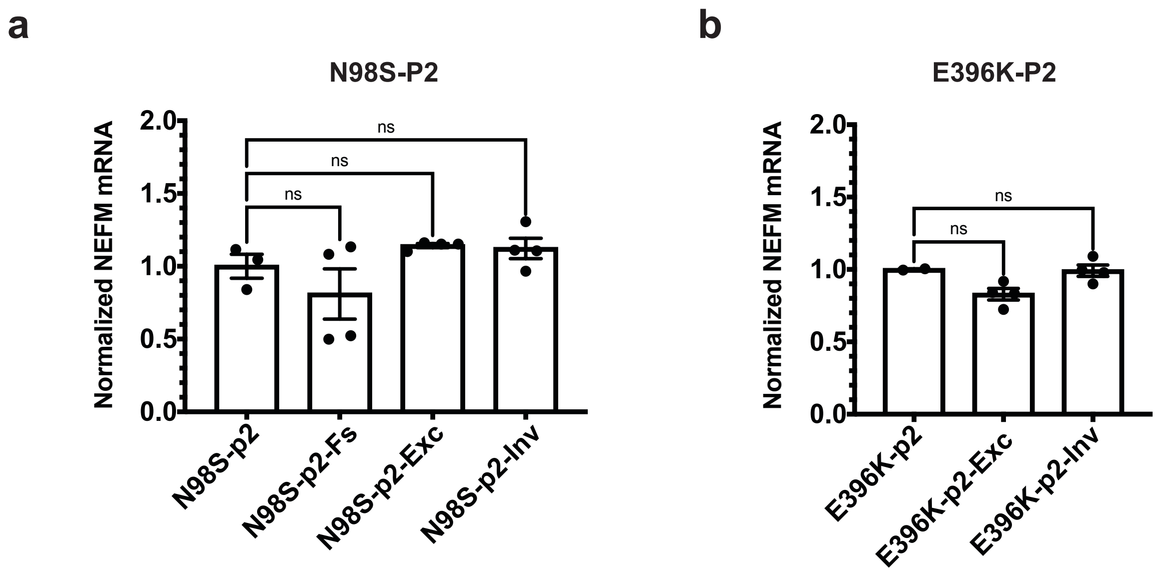

Figure S9: Total *NEFM* expression in edited i^{3}LMNs. Edited iPSCs from **(a)** N98S-P2 and **(b)** E396K-P2 were differentiated into i^{3}LMNs, and RNA was extracted on day 7. Total *NEFM* expression was measured by quantitative RT-ddPCR relative to *GAPDH* and normalized to unedited controls. Bar graphs represent mean +/- S.E.M. of biological replicates. ns $p \geq 0.05$.

bioRxiv preprint doi: https://doi.org/10.1101/2024.12.20.629813; this version posted December 22, 2024. The copyright holder for this preprint (which was not certified by peer review) is the author/funder, who has granted bioRxiv a license to display the preprint in perpetuity. It is made available under aCC-BY 4.0 International license.

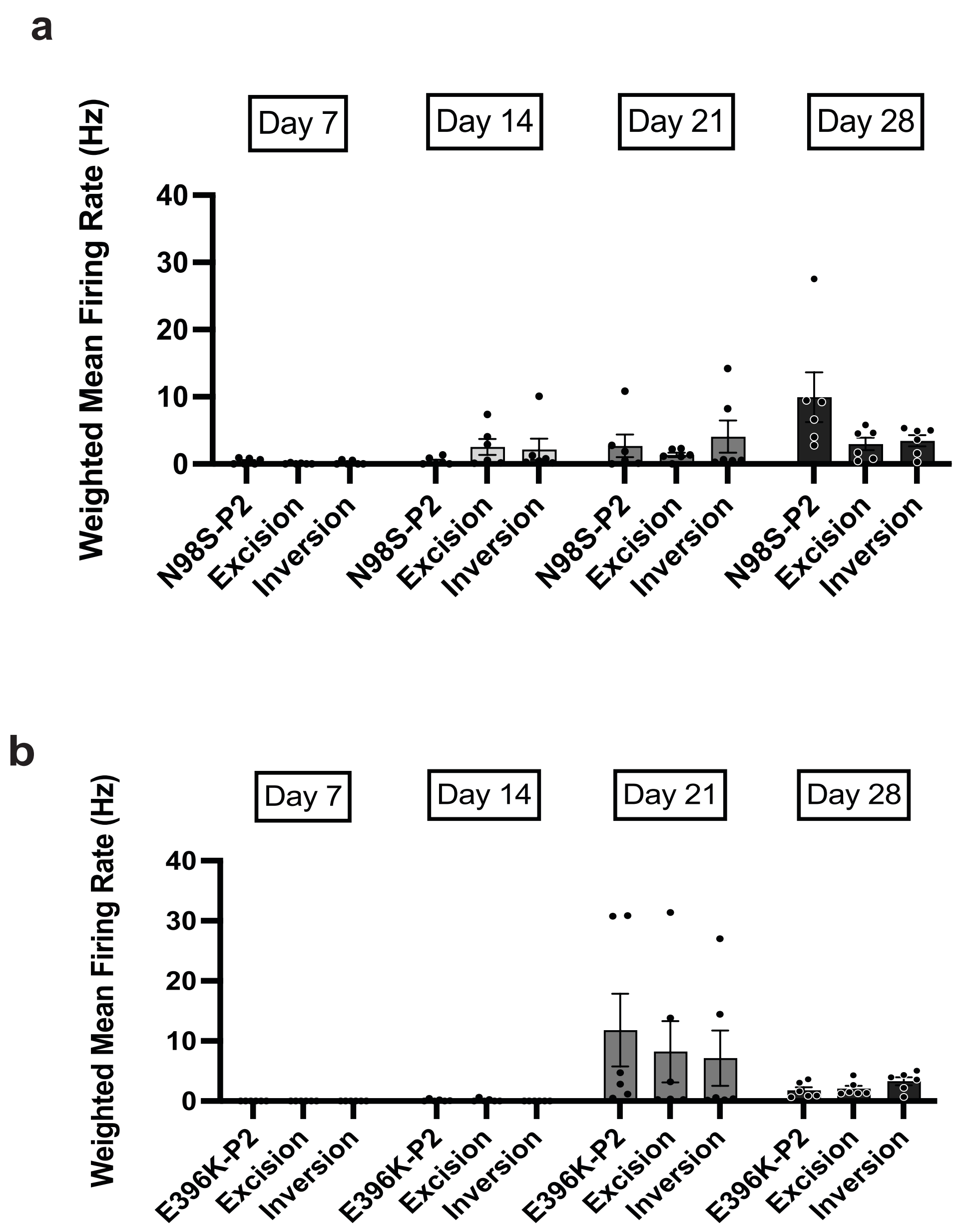

Figure S10: Total *NEFL* gene excision and inversion do not impact i^{3}LMN electrophysiology. Multi-electrode array was conducted to measure spontaneous electrical activity at days 7, 14, 21, and 28 for **(a)** N98S-P2 and (b) E396K-P2 series of edited i^{3}LMN. Each data point represents the mean weighted firing rate from one biological replicate. Two-way ANOVA demonstrated a statistically significant effect of the differentiation day ($p < 0.001$) but no significant difference between cell lines ($p > 0.05$). Bar graphs represent mean +/- S.E.M of biological replicates.

bioRxiv preprint doi: https://doi.org/10.1101/2024.12.20.629813; this version posted December 22, 2024. The copyright holder for this preprint (which was not certified by peer review) is the author/funder, who has granted bioRxiv a license to display the preprint in perpetuity. It is made available under aCC-BY 4.0 International license.

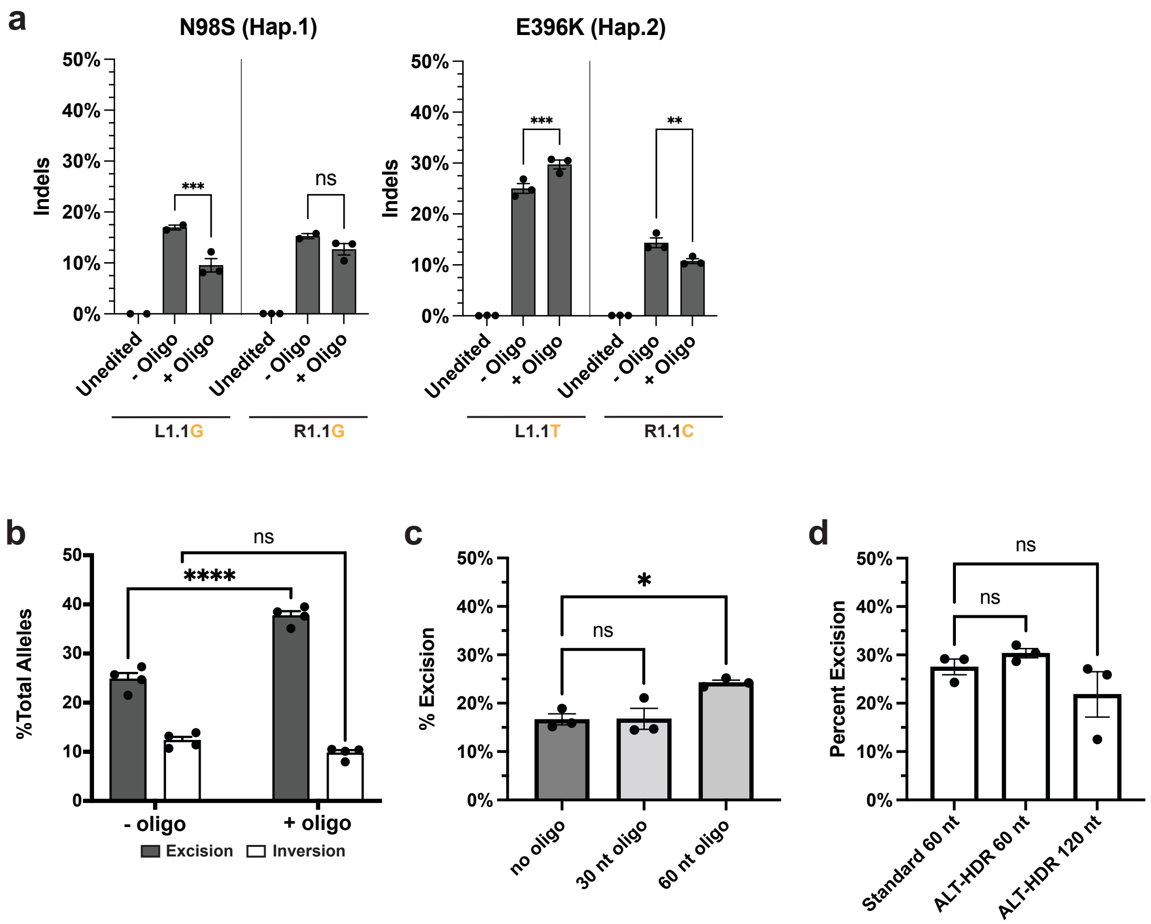

Figure S11: Effect of bridging oligos on editing outcomes. (a) N98S and E396K iPSCs were transfected with L1.1 and R1.1 RNP with and without the addition of a 60 nt single-strand oligonucleotide donor (ssODN). PCR amplicons spanning L1.1 and R1.1 target sites were analyzed by NGS amplicon sequencing to quantify indels on alleles that were not edited by excision or inversion. **(b)** N98S-P2 iPSCs were transfected with L1.1 + BA1 RNP with and without an excision bridging ssODN. Excision and inversion frequency were measured by ddPCR. **(c-d)** E396K-P2 iPSCs were transfected with L1.1 + R1.1 RNP and ssODN of different lengths and chemistries. Excision was measured by ddPCR. Comparison of 30 and 60 nt unmodified ssODN shown in (c). Comparison of unmodified (60 nt) and ALT-HDR modified (60 and 120 nt) ssODN shown in (d). For all experiments, gDNA was collected four days after nucleofection. Bar graphs represent mean +/- S.E.M. of replicate transfections. * $p < 0.05$, ** $p < 0.01$, *** $p < 0.001$, **** $p < 0.0001$, ns $p > 0.05$.

bioRxiv preprint doi: https://doi.org/10.1101/2024.12.20.629813; this version posted December 22, 2024. The copyright holder for this preprint (which was not certified by peer review) is the author/funder, who has granted bioRxiv a license to display the preprint in perpetuity. It is made available under aCC-BY 4.0 International license.

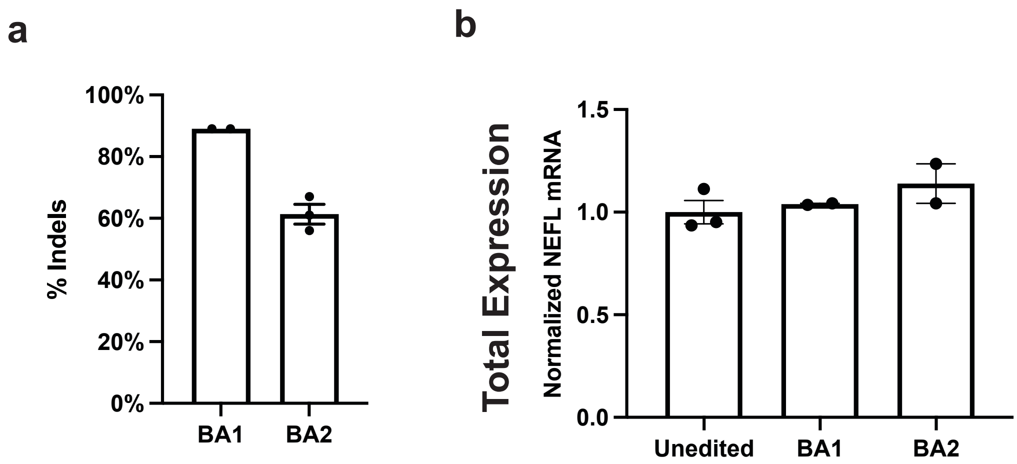

Figure S12: Evaluation of indels produced by biallelic gRNAs targeting *NEFL* intron 1. (a) Quantification of indels induced by BA1 and BA2 gRNAs as measured by Sanger sequencing and ICE analysis. **(b)** Total *NEFL* expression measured by quantitative RT-ddPCR, normalized to *GAPDH*. Bar graphs represent the mean of replicate transfections (a) or replicate differentiations (b) +/- S.E.M.

bioRxiv preprint doi: https://doi.org/10.1101/2024.12.20.629813; this version posted December 22, 2024. The copyright holder for this preprint (which was not certified by peer review) is the author/funder, who has granted bioRxiv a license to display the preprint in perpetuity. It is made available under aCC-BY 4.0 International license.

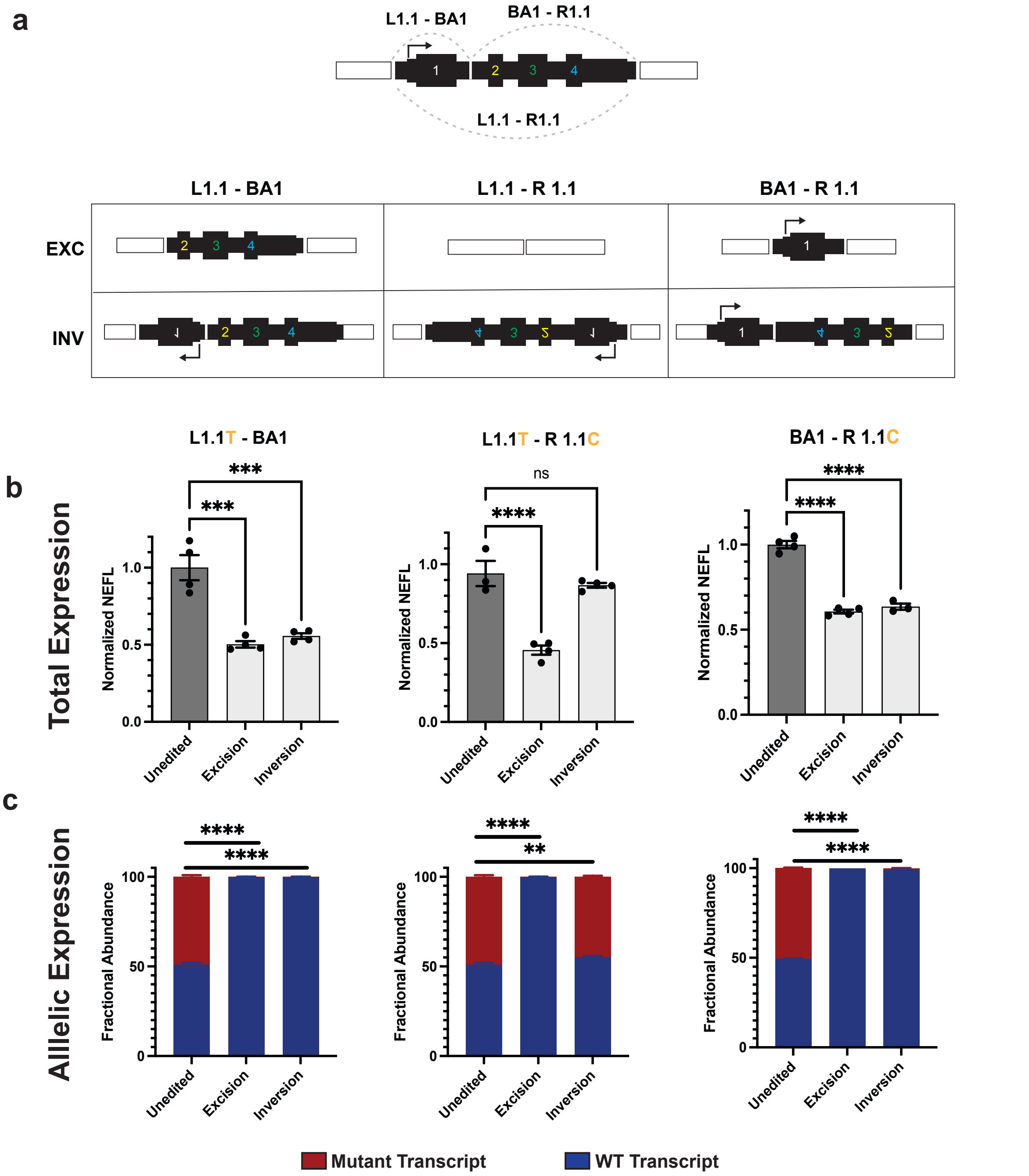

Figure S13: Clonal analysis of editing outcomes reveals variable contributions of excision and inversion to gene inactivation. (a) Schematic of predicted editing outcomes with various combinations of L1.1, R1.1 and intronic biallelic (BA1) gRNAs. E396K-P2 was transfected with each RNP pair and clonal iPSC lines were isolated with each predicted outcome and differentiated into i^{3}LMNs. RNA was isolated on day 7 and measured by quantitative RT-ddPCR. **(b)** Total *NEFL* expression relative to *GAPDH* and normalized to unedited control. **(c)** Relative allelic expression via allele discrimination ddPCR using a heterozygous SNP in the 3' UTR (rs2976439). Bar graphs represent the mean +/- S.E.M. of biological replicates. ** $p < 0.01$, *** $p < 0.001$, **** $p < 0.0001$, ns $p > 0.05$.

bioRxiv preprint doi: https://doi.org/10.1101/2024.12.20.629813; this version posted December 22, 2024. The copyright holder for this preprint (which was not certified by peer review) is the author/funder, who has granted bioRxiv a license to display the preprint in perpetuity. It is made available under aCC-BY 4.0 International license.

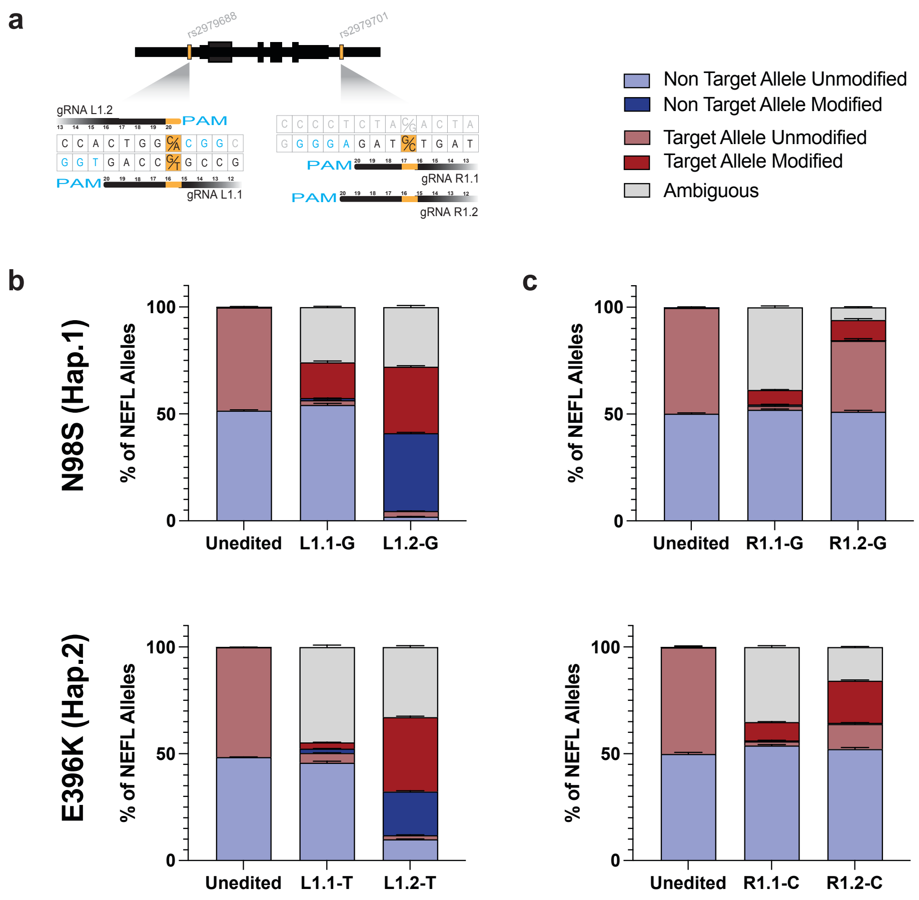

Figure S14: Analysis of allele-specific editing with single gRNA. (a) Schematic of sequences targeted by SNP-specific gRNAs with variants in orange and the associated PAM in blue. **(b-c)** N98S-P2 and E396K-P2 iPSC were transfected with the indicated allele-specific gRNA-HiFiCas9 RNPs followed by quantification of editing outcomes by NGS amplicon sequencing and CRISPResso2 analysis. Ambiguous editing events cannot be assigned to either allele due to the deletion of the variant nucleotide. Bar graphs represent the mean +/- S.E.M. of triplicate transfections.

bioRxiv preprint doi: https://doi.org/10.1101/2024.12.20.629813; this version posted December 22, 2024. The copyright holder for this preprint (which was not certified by peer review) is the author/funder, who has granted bioRxiv a license to display the preprint in perpetuity. It is made available under aCC-BY 4.0 International license.

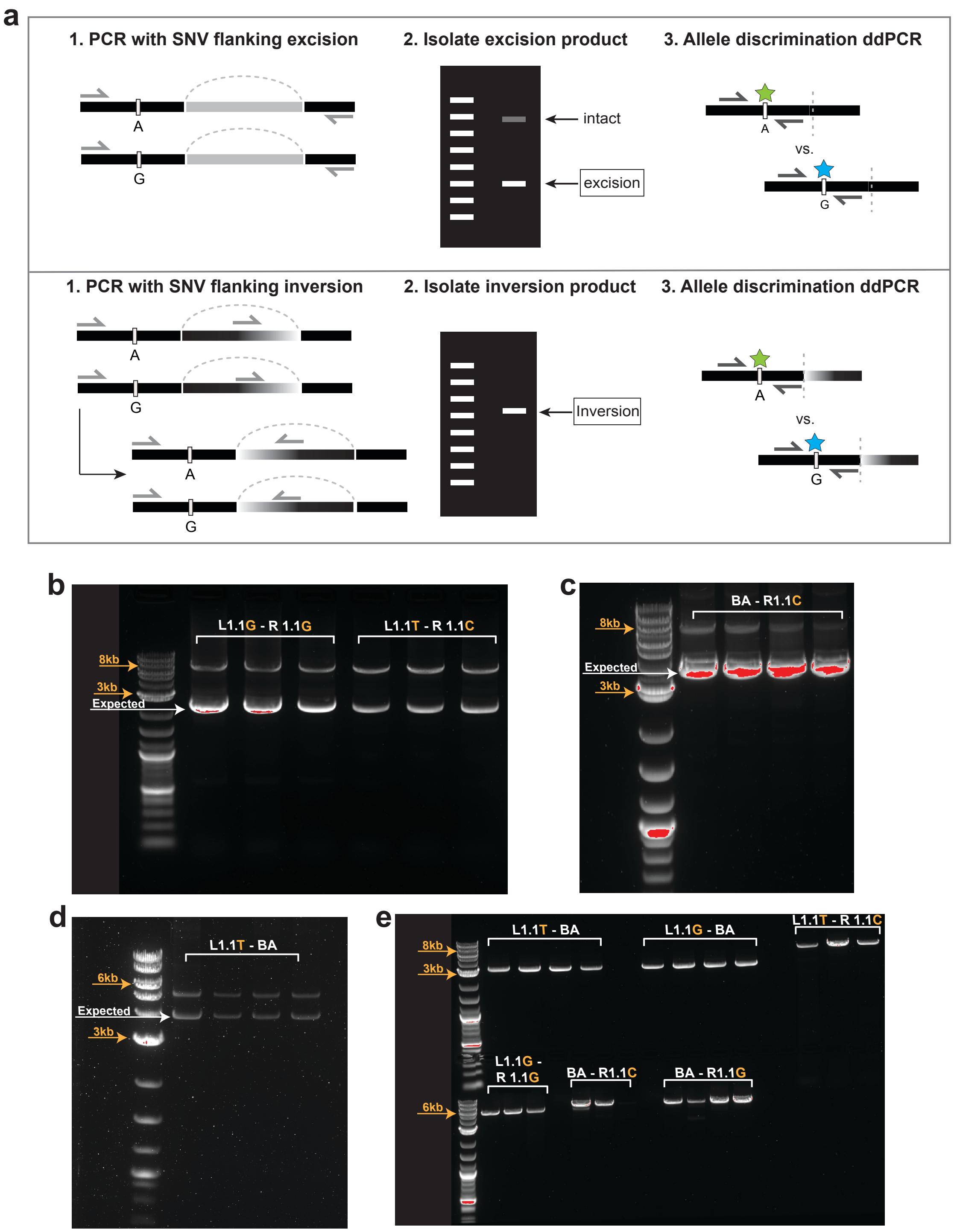

Figure S15: Multi-step PCR/ddPCR assay to measure allele-specificity of excision and inversion. (a) Assay workflow 1: Schematic of PCR designed to amplify excised or inverted alleles with flanking heterozygous variant; 2: Gel purification to isolate excision or inversion products 3: Schematic of allele discrimination ddPCR used to quantify specificity. **(b-d)** Examples of gel electrophoresis of PCR products used for excision specificity. **(e)** Examples of gel electrophoresis of PCR products used for inversion specificity.

bioRxiv preprint doi: https://doi.org/10.1101/2024.12.20.629813; this version posted December 22, 2024. The copyright holder for this preprint (which was not certified by peer review) is the author/funder, who has granted bioRxiv a license to display the preprint in perpetuity. It is made available under aCC-BY 4.0 International license.

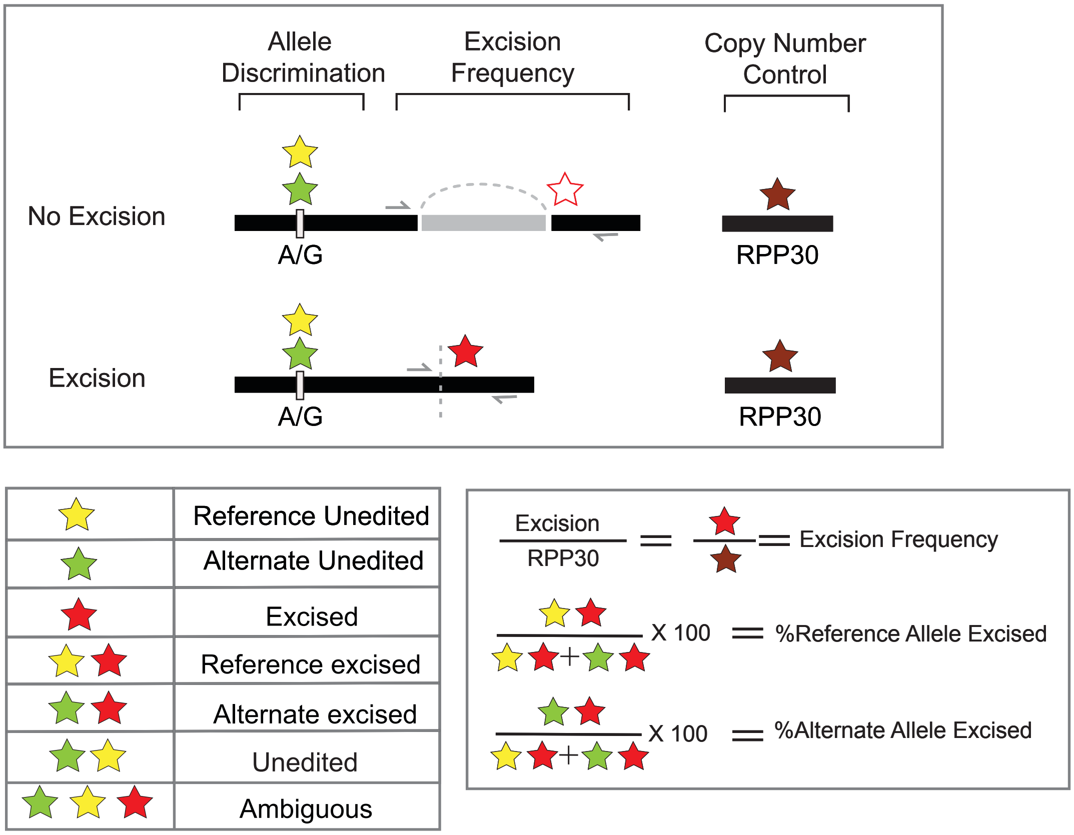

Figure S16: Single-step assay to measure excision frequency and specificity via multiplexed four-color digital PCR. Top: Schematic of multiplexed assay design with four probes (Green = FAM, Yellow = Hex, Red = ROX, Maroon = Cy5). **Bottom-left:** Partition occupancy observed for excision specificity measurements. **Bottom-right**: Calculation used to quantify excision specificity and frequency.

bioRxiv preprint doi: https://doi.org/10.1101/2024.12.20.629813; this version posted December 22, 2024. The copyright holder for this preprint (which was not certified by peer review) is the author/funder, who has granted bioRxiv a license to display the preprint in perpetuity. It is made available under aCC-BY 4.0 International license.

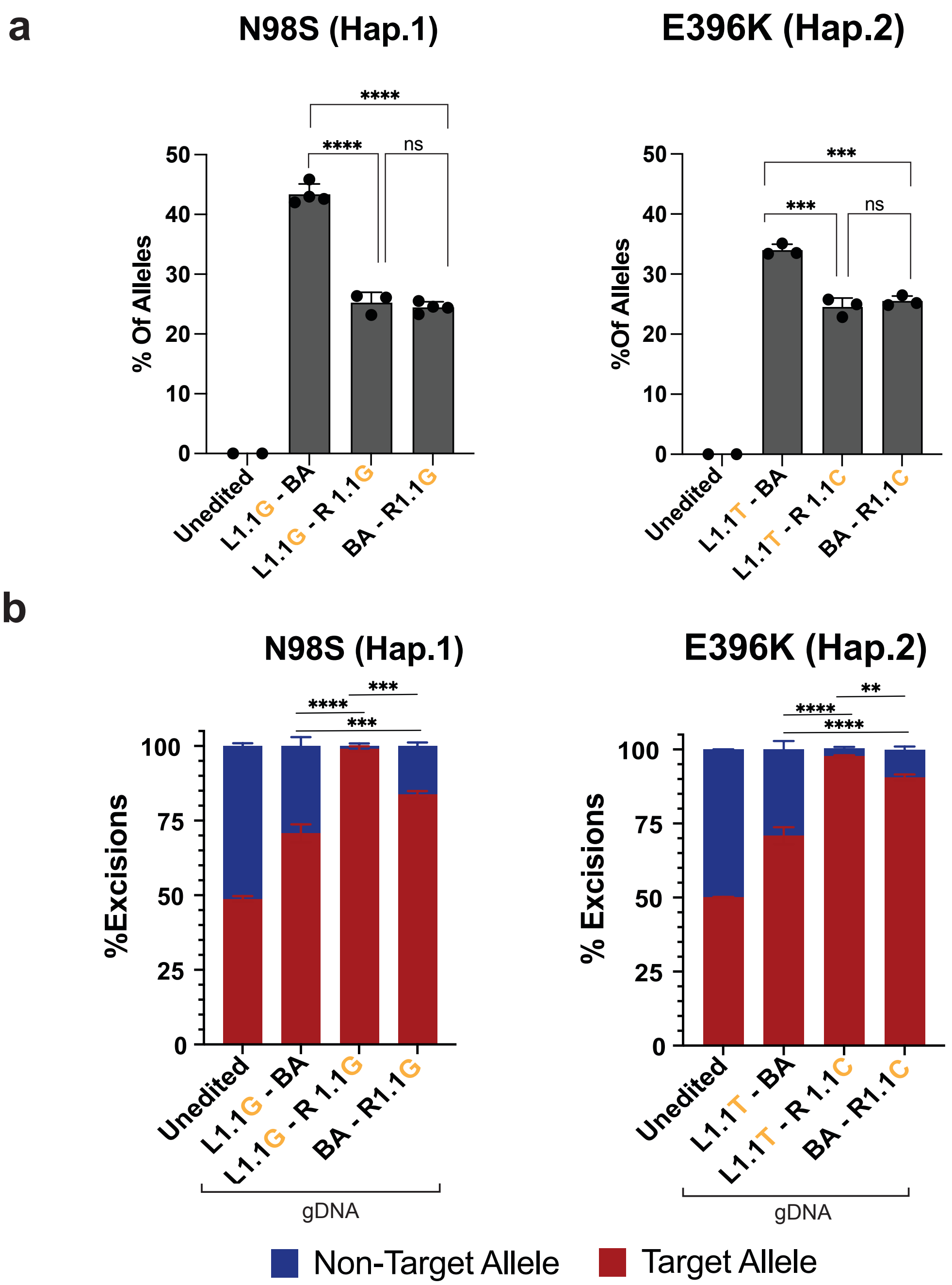

Figure S17: Quantification of excision frequency and specificity via multiplexed four-color digital PCR. (a) Excision frequency as measured by the ratio of excision (ROX+) signal over RRP30 (Cy5+) signal. **(b)** Excision specificity measured via dPCR allele-discrimination assay for a heterozygous SNP (rs2979685, ref = HEX, alt = FAM) located 5' of *NEFL*. Unedited control represents the fractional abundance of HEX vs. FAM in ROX-negative partitions to demonstrate equal abundance of the two alleles at baseline, while edited samples represent the fractional abundance of HEX vs. FAM in ROX-positive partitions that contain an excision event. Bar graphs represent mean +/- S.E.M. of replicate transfections. ** $p < 0.01$, *** $p < 0.001$, **** $p < 0.0001$, ns $p > 0.05$.

bioRxiv preprint doi: https://doi.org/10.1101/2024.12.20.629813; this version posted December 22, 2024. The copyright holder for this preprint (which was not certified by peer review) is the author/funder, who has granted bioRxiv a license to display the preprint in perpetuity. It is made available under aCC-BY 4.0 International license.

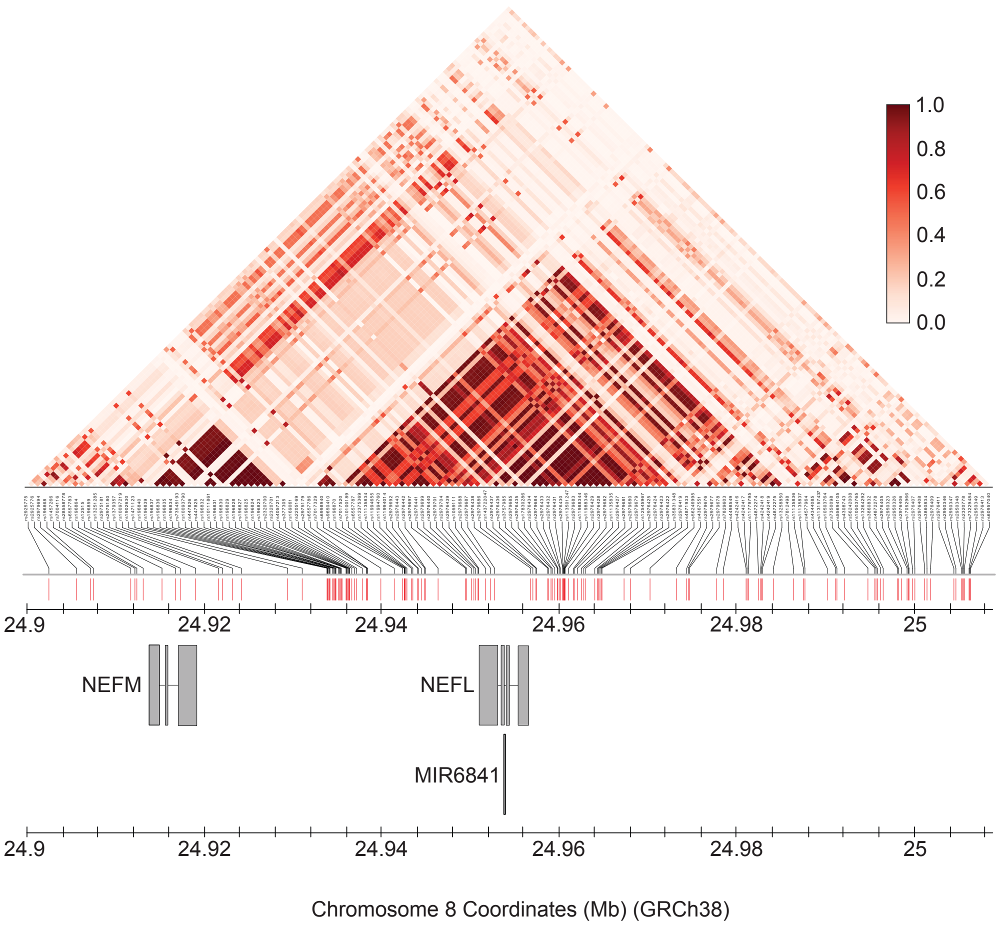

Figure S18: Linkage measured as R^2 for *NEFL* and 50 kb of flanking DNA on each side. Linkage values are shown for all SNPs with allele frequency between 0.1 and 0.9 in the region and their position relative to genes in the region is shown below. R^2 values were downloaded from LDLink.

bioRxiv preprint doi: https://doi.org/10.1101/2024.12.20.629813; this version posted December 22, 2024. The copyright holder for this preprint (which was not certified by peer review) is the author/funder, who has granted bioRxiv a license to display the preprint in perpetuity. It is made available under aCC-BY 4.0 International license.

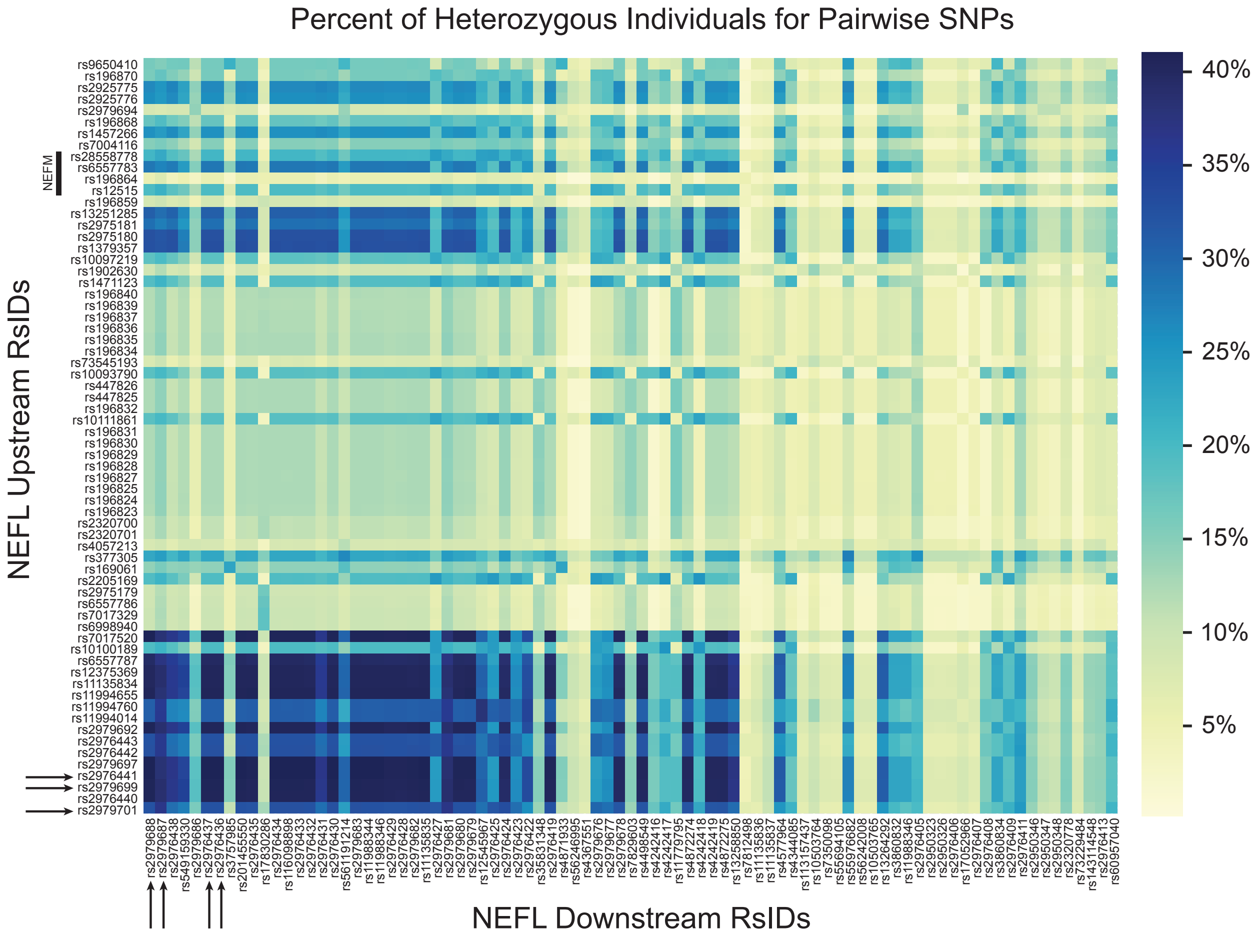

Figure S19: Percentage of individuals heterozygous for pairwise SNPs flanking *NEFL*. Variant data from 1000 Genomes phase 3, starting with minor allele frequency 0.1-0.9 in global population. Horizontal axis indicates SNPs downstream (3') of *NEFL* increasing in linear distance from left to right. Vertical axis indicates SNPs upstream (5') of *NEFL* increasing in linear distance from bottom to top. The location of *NEFM* (upstream of *NEFL*) is annotated with a line along the vertical axis. Darker blue colors indicate a higher percentage of heterozygous individuals. Arrows indicate the SNPs targeted in this study.
